# Induction of Tolerogenic Dendritic Cells by Endogenous Biomolecules: An Update

**DOI:** 10.3389/fimmu.2018.02482

**Published:** 2018-10-26

**Authors:** Urban Švajger, Primož Rožman

**Affiliations:** ^1^Department for Therapeutic Services, Blood Transfusion Centre of Slovenia, Ljubljana, Slovenia; ^2^Faculty of Pharmacy, University of Ljubljana, Ljubljana, Slovenia

**Keywords:** tolerogenic dendritic cell, cytokines, biomolecules, growth factors, complement system, lectins, hormones, tolerance

## Abstract

The importance of microenvironment on dendritic cell (DC) function and development has been strongly established during the last two decades. Although DCs with general tolerogenic characteristics have been isolated and defined as a particular sub-population, it is predominantly their unequivocal biological plasticity, which allows for unparalleled responsiveness to environmental ques and shaping of their tolerogenic characteristics when interacting with tolerance-inducing biomolecules. Dendritic cells carry receptors for a great number of endogenous factors, which, after ligation, can importantly influence the development of their activation state. For this there is ample evidence merely by observation of DC characteristics isolated from various anatomical niches, e.g., the greater immunosuppressive potential of DCs isolated from intestine compared to conventional blood DCs. Endogenous biomolecules present in these environments most likely play a major role as a determinant of their phenotype and function. In this review, we will concisely summarize in what way various, tolerance-inducing endogenous factors influence DC biology, the development of their particular tolerogenic state and their subsequent actions in context of immune response inhibition and induction of regulatory T cells.

## Introduction

Dendritic cells (DCs) comprise a heterogenous and specialized immune cell subset with the main role of sampling and presenting both endogenous and foreign antigens (Ags) to cells of the adaptive immune system. In addition to their exceptional antigen-presenting capacity, they also possess extensive functional plasticity that enables DCs to initiate and control both immunogenic and tolerogenic immune responses (1, 2). The capacity of DCs to induce either immunity or tolerance is largely dictated by their activation state, which in turn is greatly determined by their specific microenvironment. We now know that DCs are equipped with numerous surface and intracellular receptors which recognize danger- and pathogen-related signals, as well as inhibitory signals, which can trigger their tolerogenic activation state (3). Considering their life-cycle, immature DCs are mainly found near body surfaces in physiological conditions, where their main task is to sample and process Ags for future presentation to Ag-specific T cells. Immature DCs express low levels of co-stimulatory molecules and produce little or no pro-inflammatory cytokines. The immature state alone can induce T cell anergy or even *de novo* induction of regulatory T cells (Tregs), due to Ag-presentation in the absence of signal 2 (co-stimulatory molecules), or signal 3 (soluble cytokines) delivery. This can also be referred to as passive tolerance induction. In the case of an encounter with pathogen-associated molecular patterns (PAMPs) or danger-associated molecular patterns (DAMPs), DCs reach their opposite activation state, termed mature DCs, which migrate to adjacent lymph nodes with an extensive capacity to induce effector T cells. In the case of partial maturation (e.g., exposure to TNF-α for a limited period of time), the DCs can obtain a so-called semi-mature activation state. This means there is either a lack of certain phenotypic markers or a lower production of pro-inflammatory cytokines, which can lead to tolerogenic outcome after interaction with responding T cells (4), but does not exclude the potential of generating effector responses in certain instances (5). Tolerogenic DCs (TolDCs) on the other hand are induced by numerous immunosuppressive agents which can represent cytokines such as interleukin (IL)-10 or transforming growth factor (TGF)-β, endogenous immunosuppressants such as glucocorticoids, as well as several synthetic immunosuppressive drugs (e.g., rapamycin, aspirin), natural products (e.g., curcumin, resveratrol) and others (6, 7). If one was to search for reason why TolDCs are much more efficient in inducing tolerogenic responses in comparison to immature DCs, it is the presence of elements of active tolerance-induction (surface inhibitory molecules, immunosuppressive cytokines), which are expressed on TolDCs in an extensive manner.

One of the first reports of using an immunosuppressive agent to induce an *in vitro* tolerogenic state in DCs is that of Steinbrink et al., where they showed that IL-10-treated DCs display significantly reduced allo-stimulatory potential, a low expression level of CD86 and T cell anergy (8). A few years later it was shown that a similar effect can be achieved using small molecule immunosuppressants, namely corticosteroids (9) or the active form of vitamin D (vit D_3_) (10). Since then, a great number and variety of biomolecules or synthetic drugs have been shown to affect different stages of the DC life-cycle in a way that inhibits their maturation potential or even induces tolerogenic properties. Several good quality reviews have also been written on this subject, particularly on the subject of pharmacological agents. We refer the reader to these manuscripts in order to get a more detailed insight on the background of TolDC induction (11–14). However, in recent years we have witnessed several reports highlighting the tolerogenic role of several endogenous biomolecules not previously discussed in detail (Table 1). In this review, we will focus mainly on these novel findings with the goal of contributing an up-date on previous discussions.

**Table 1:** The effects of various tolerogenic biomolecules on DC phenotype and function.

**Biomolecules**	**Effect on DC characteristic/subsequent T cell response**	**References**
**Cytokines**
IL-10	↓ maturation, DC-10, ↑ inhibitory molecules, T cell anergy, Treg induction	(8, 15, 57–61)
TGF-β	CD8^+^ Treg induction, EAE attenuation	(17, 18)
IFN-α	Semi-mature, ↑IL-10 production, T cell apoptosis, Tr1 induction	(19)
TNF-α	Semi-mature, FoxP3^+^ Treg induction	(20)
VIP	↓ maturation, ↑ IL-10 production, CD4^+^/CD8^+^ Treg induction	(21, 22)
IL-16+thrombopoietin	↑ ILT-2/ILT-3/ILT-4 expression, T cell anergy	(23)
IFN-λ	↓ co-stimulatory molecules, FoxP3^+^ Treg expansion	(24)
IFN-γ	↑ IDO competence, ↑ ILT-4/HLA-G expression, ↓ CD8^+^ cytotoxic responses	(26, 29–37)
IL-37	↓ maturation *in vivo*, ↑ IL-10 production, ↓ allo-stimulatory capacity, Treg induction	(42, 43)
IL-35	↓ co-stimulatory capacity, ↑ IL-10 production, ↓ monocyte-to-DC differentiation	(44–47)
IL-27	↑ PDL-1 and CD39 expression, ↑ extracellular ATP catabolism, ↓ Th9 differentiation	(52–55)
LECTINS^*^
DC-SIGN	↓ maturation, modulation of PRR signaling, ↑ IL-10 production	(64–67)
Galectin-1	↑ tolerogenic characteristics via IL-27 and IL-10, ↓ IL-12p70 production	(74, 78)
Siglec-E	↓ maturation, inhibition of TLR-activated Nf-κB	(80, 84)
Siglec-H	inhibition of T cell responses via pDCs in EAE	(82)
Siglec-1	semi-mature phenotype of pDCs, ↓ IFN-α production	(83)
**Complement system**
C1q	Resistance to maturation, ↓ co-stimulatory molecules, ↓ allo-stimulatory capacity	(88, 89)
C4BP α_7_β_0_	Semi-mature state, ↓ CD80/CD83/CD86 expression, ↑ IL-10 production, FoxP3^+^ Treg induction	(92, 93)
Factor H	↓ CD40/CD80/CD86/MHC-II expression, ↑ IL-10 production	(103, 106)
**Growth factors**
VEGF	↓ maturation, ↓ T cell-stimulatory capacity, TLR-4 modulation via Nrp-1	(109, 111–113)
PIGF	↓ CD40/CD80/CD83/CD86/MHC-II expression, ↓ IL-8, IL-12p70, TNF-α production	(114)
HGF	High IL-10/IL-12 ratio, ↑ ILT-3 expression, FoxP3^+^ Treg induction	(119–121, 123)
Adrenomedullin	Semi-mature state, ↑ IDO-competence, FoxP3^+^ and IL-10^+^ Treg induction	(131, 132)
**Hormones**
Glucocorticoids	↓ monocyte-to-DC differentiation, resistance to maturation	(134–147)
vit D_3_	↓ monocyte-to-DC differentiation, ↑ ILT-3 and PDL-1 expression	(148–152)
hCG	↑ IL-10 production, ↓ Ag-specific T cell proliferation, *in vivo* Treg induction	(154, 156, 157)
Progesterone	↓ T-cell stimulatory capacity *in vivo*	(158, 159)
**Neurotransmitters**
Serotonin	↓ monocyte-to-DC differentiation, ↑ IL-10/IL-12 ratio, ↓ CXCL-10 production	(164–166)
Histamine	↓ IL-12 production, ↓ CXCL-10 production, ↑ Th2 polarization,	(167–172)
Adrenaline	↑ IL-10 production, ↓ IL-6, IL-12, IL-23 production, FoxP3^+^ Treg induction	(175)

* *Present on DC surface*.

## Cytokines

More than 20 years have now passed since Steinbrink et al. have shown that the treatment of immature, monocyte-derived DCs with IL-10 results in resistance to maturation stimuli and the acquisition of functional tolerogenic properties (8). A few years later, the same group demonstrated that IL-10-treated DCs induce both CD4^+^ and CD8+ anergic T cells with regulatory functions (15). Soon after, another immunosuppressive cytokine, namely transforming growth factor (TGF)-β, was shown to induce tolerogenic antigen-presenting cells (APCs). Their adoptive transfer to mice with experimental autoimmune encephalomyelitis (EAE) attenuated disease severity via the induction of CD8+ regulatory T cells (16). In experimental diabetes setting, TGF-β-treated DCs conferred islet-specific protection via the induction of Fox P3^+^ Tregs (17, 18). At about the same time, several other biomolecules were identified as having the capacity to induce DC tolerance such as interferon (IFN)-α (19), TNF-α (20), vasoactive intestinal peptide (VIP) (21, 22), combination of IL-16 and thrombopoietin (23) and IFN-λ (24). It must be emphasized that the use of pro-inflammatory cytokines such as TNF-α and IFN-α to achieve DC tolerance can be specific to particular study designs and experimental models, since immunogenic maturation can also be achieved using these same cytokines (5, 25).

**Figure 1:**
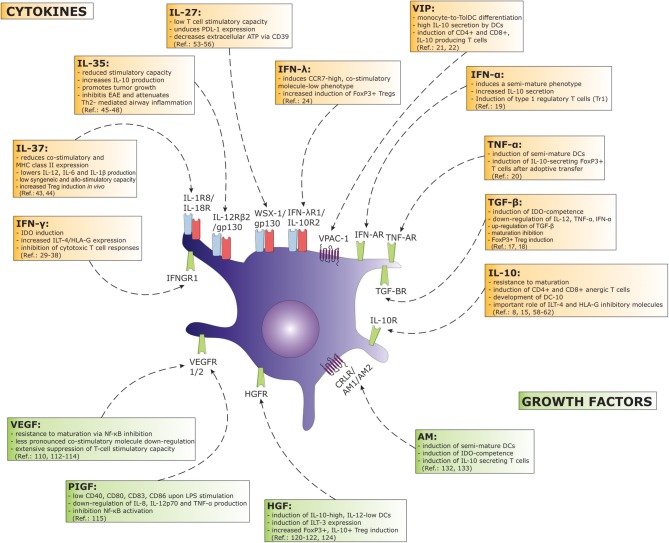
A great number of cytokines and growth factors exert a considerable tolerogenic effect in terms of DC function. Major effects on DC biology concerning a particular factor are depicted on the figure. Arrows associate cytokine or growth factor with their corresponding receptor found on DCs. (AM, adrenomedullin; HGF, hepatocyte growth factor; IDO, indoleamine-2,3-dioxygenase; IFN, interferon; IL, interleukin; ILT, immunoglobulin-like transcript; Nf-κB, nuclear factor κB; PDL, programmed death ligand; PIGF, placental growth factor; TGF, transforming growth factor; TNF, tumor necrosis factor; VEGF, vascular endothelial growth factor; VIP, vasoactive intestinal peptide).

In more recent years we have witnessed several additions to the understanding of how various other biomolecules can influence DC biology in an immunosuppressive fashion (Figure 1). Interestingly, IFN-γ, a well-known Th1-signature cytokine has been associated with DC tolerance in specific settings (26). As regards to DC biology, its role as a priming agent has been firmly established, where it can greatly induce both maturation-associated phenotypic markers and IL-12p70 production when combined with either CD40 ligand (CD40L) or toll-like receptor (TLR) activation (27, 28). However, the pleiotropic nature of IFN-γ has been demonstrated in many experimental models, and the mechanisms regarding its anti-inflammatory actions are beginning to emerge. Following DC maturation and extensive IL-12 production, their stimulatory capacity can be reduced over time in a phenomenon known as “DC exhaustion.” Interferon-γ plays a role in this process by the induction of indoleamine-2,3-dioxygenase (IDO), a tryptophan-catabolizing enzyme known for its immunoregulatory function (29). In the absence of maturation stimuli, IFN-γ has been shown to be a crucial inducer of IDO-competence and able to generate DCs with regulatory properties in an IFN-γ-rich environment (30). The effect of tryptophan catabolites, namely kynurenines, can spread the tolerogenic function beyond cell contact to otherwise immunogenic DCs, as was shown in transwell experiments. The tolerogenic function of DCs expressing IFN-γ-induced IDO can be seen in reduced T cell proliferation (31) and the induction of Tregs (32). It was also shown that IDO, induced in DCs after contact with apoptotic cells, is the result of the autocrine production of IFN-γ, the blockade of which diminishes IDO expression (33). The context-specific role of IFN-γ was recently demonstrated by our group, where we investigated the effects of an IFN-γ-rich environment on the DC inhibitory phenotype. Particularly at high concentrations, IFN-γ did not induce extensive DC maturation, but strongly up-regulated inhibitory molecules of HLA-G and the immunoglobulin-like transcript (ILT)-4 (34). Such IFN-γ-high DCs suppressed cytotoxic T cell responses with a down regulation of T cell proliferation and granzyme B expression. This effect was IDO-independent and could be reversed by HLA-G blocking mAbs. The tolerogenic role of IFN-γ was frequently described *in vivo*. For example, its disease-attenuating effects have been described in EAE, experimental arthritis, as well as colitis (35–37). Furthermore, there are numerous reports describing an immunosuppressive role of IFN-γ in models of graft-vs.-host-disease (GvHD)(26). An important characteristic when observing the immunoregulatory effects of IFN-γ *in vivo* are its paradoxical actions, where it can aggravate disease severity in some cases, while attenuating disease progression in others, e.g., in EAE. This is frequently dependent on the time course of disease (e.g., IFN-γ treatment/blockade before or after disease onset). In detail mechanisms regarding these and several other phenomena of IFN-γ have been recently discussed by Svajger and colleagues and we refer the reader to this review for further reading (26).

Interleukin-37, an IL-1 family member, was discovered in the year 2000 by several independent groups using *in silico* research of human databases (38). Initially its anti-inflammatory effects were shown in the context of innate immunity functions, demonstrating its ability to down regulate all major pro-inflammatory cytokine production and attenuating experimental lipopolysaccharide (LPS)-induced shock (39, 40). It has been suggested that the regulation of innate immunity by IL-37 requires it to bind to the IL-1R8 receptor in addition to the previously recognized IL-18R (41). More recently, the effects of IL-37 have been associated with the suppression of adaptive immune responses via the induction of TolDCs. Skin DCs in mice transduced with human IL-37b isoform displayed tolerogenic characteristics in response to a contact hypersensitivity challenge (42). Stimulation of isolated DCs with LPS showed significantly reduced MHC class II and co-stimulatory molecule expression in comparison to wild-type (WT) mice. In addition, the production of IL-1β, IL-6, and IL-12 was reduced, while IL-10 production increased. In terms of function, DCs from IL-37 transgenic mice displayed a reduced stimulatory capacity regarding both syngeneic and allogeneic T cells and showed enhanced induction of Tregs *in vitro* (42). In a recent study, bone marrow-derived DCs treated with IL-37 and troponin I showed a tolerogenic phenotype with an increased expression of IL-10 and IDO mRNA. Such TolDCs improved cardiac remodeling after myocardial infarction in a mouse model by establishing a tolerogenic response and inducing Treg development (43).

Another newly discovered cytokine, a heterodimer between Epstein-Barr-virus-induced protein 3 (EBI3) and IL-12α, termed IL-35, has been identified as having important regulatory properties, initially in the context of Fox P3^+^ Tregs (44). The signaling pathway of IL-35 is initiated through a unique heterodimer of receptor chains, namely the IL-12Rβ2 and gp130, or via homodimers of each chain (45). Later on, its role in regulating the function of murine CD8α+ DCs was established in a study using transduced DC cell lines with constitutive IL-35 expression. Besides the direct regulatory effects of secreted IL-35 on T cell function both *in vitro* and *in vivo*, it also exerted autocrine effects inducing a tolerogenic DC phenotype characterized by reduced co-stimulatory capacity and increased IL-10 production (46). Vaccination of mice with IL-35-expressing DCs promoted melanoma and colon carcinoma growth, while at the same time prevented the development of EAE. The *in vivo* inhibitory effect on DC function was also demonstrated in an ovalbumin-induced asthma model. Intraperitoneal IL-35 administration during the allergen sensitization phase was efficient in attenuating allergic airway inflammation with reduced Th2 cytokine signature. Importantly, IL-35 treatment reduced the development of tissue-present monocytes to inflammatory DCs (47). Although IL-35 production was specifically shown for Tregs, Dixon et al. have recently shown that human, monocyte-derived DCs produce IL-35 upon treatment with Dexamethasone (48). The production of IL-35 was significant for optimal DC tolerogenic function.

Similarly to IL-35, another IL-12 family member, IL-27, is a heterodimeric cytokine composed of EBI3 and IL-27p28 (49). It mediates its biological functions through a high-affinity receptor complex composed of WSX-1 (IL-27 receptor α subunit) and gp130, differentially expressed on immune cells (50). Interleukin-27 is known by its antagonistic function on Th17 and the capacity to generate IL-10 producing, type 1 regulatory T cells (Tr1) (51). Dendritic cells have been shown to be an important source of IL-27, a mechanism which allows DC-mediated induction of Tr1 cells (52). Interestingly, IFN-γ treatment of DCs promotes IL-27 secretion (53). In a similar manner, IFN-γ-induced IL-27 production by DCs also inhibits Th9 cell differentiation (54). Besides its modulatory effects on T cells, IL-27 also directly affects DC function. A study on *in vitro* monocyte-derived DCs and blood DCs demonstrated the low T cell-stimulatory capacity of IL-27-treated DCs, along with low cytokine production by stimulated T cells. The DCs' regulatory function was dependent on IL-27-induced expression of PDL-1 (55). The regulatory function of IL-27-treated DCs was also evident in a mouse EAE model, where suppression of disease was mediated via the IL-27-induced expression of immunoregulatory molecule CD39 and the subsequent decrease in extracellular ATP concentration (56).

Finally, well-established immunosuppressive cytokines, such as IL-10, have been revisited, in terms of tolerogenic DC induction, in more recent times. In 2010, Gregori et al. identified and characterized a new subset of high IL-10-producing TolDCs, termed DC-10. According to their report, DC-10 are present *in vivo* and can be described as CD1a^−^, CD1c^−^, CD14^+^, CD16^+^, CD11c^+^, CD11b^+^, CD83^+^, HLA-DR^+^. Interestingly, these cells also express high levels of co-stimulatory molecules CD40, CD86, and up-regulate CD80 upon activation (57). Nevertheless, DC-10 also show abundant expression of inhibitory molecules ILT-2, ILT-3, ILT-4, and HLA-G. The *in vitro* counterparts of DC-10 could be differentiated from monocytes in the presence of IL-4 and IL-10. The immunosuppressive effect of IL-10 on DCs has already been demonstrated on many occasions. As far back as 1998, Allavena et al. demonstrated that the addition of IL-10 to monocyte-to-DC differentiation cultures results in the “inhibition” of DC maturation and the preferential generation of cells with a macrophage-like phenotype (58). Other, earlier studies also reported on the reduced allo-stimulatory capacity of IL-10-treated DCs, although there were also contradictory reports on preferential DC generation (59). The more detailed characterization by Gregori et al. allows for the understanding that, although phenotypically similar to macrophages, DC-10 possess several DC-like qualities and are therefore more likely to be regarded as such. The expression of inhibitory molecules, particularly HLA-G on DC-10 has been strongly correlated with their immunosuppressive function (60). It has been shown that DC-10 can specifically induce the generation of Tr1 cells, which was shown to require both IL-10 and the interaction between ILT-4 and HLA-G. Furthermore, Amodio et al. have shown that HLA-G expression is genetically imprinted, and therefore donor-specific, ranging from a few percent to completely positive, depending on the donor (60). Since a high expression level of HLA-G is required for efficient induction of Tr1 cells by DC-10, correct donor selection must be respected in this regard.

Additional recent data also highlight the somewhat diverse effects of IL-10 on DC biology. In a study by Kryczanowsky et al., IL-10-treatment of monocyte-derived DCs on day 5 (in the presence of maturation stimuli) resulted in two major populations defined as CD83^high^CCR7^+^ and CD83^low^CCR7^−^ DCs. The CD83^high^ DCs displayed a superior immunosuppressive function compared to that of CD83^low^. Additionally, CD83^high^ DCs possessed the capacity to migrate toward secondary lymph node chemokine CCL21 (61). Of interesting note, an alternative DC-associated immunosuppressive mechanism by CD83^high^ cells was proposed based on surface expression and secretion of CD25. Similarly to CD25-expressing Tregs, CD25 secreted by DCs can inhibit IL-2-dependent T cell proliferation. This was described previously in the context of tumor-mediated DC suppression via PGE2 (62). Lastly, the extensive tolerogenic potential of DC-10 indicates the importance of DC life-cycle stage in their tolerogenic development. Although treatment of already established immature DCs with IL-10 is known to induce tolerogenic properties and resistance to maturation, DC-10, which are generated with IL-10 present during their differentiation phase have been found superior in this aspect. It is very likely this is not merely due to prolonged IL-10 exposure but concerns more fundamental changes in cell biology development, as somewhat indicated by their unique phenotype.

## Glycan-binding lectins

**Figure 2:**
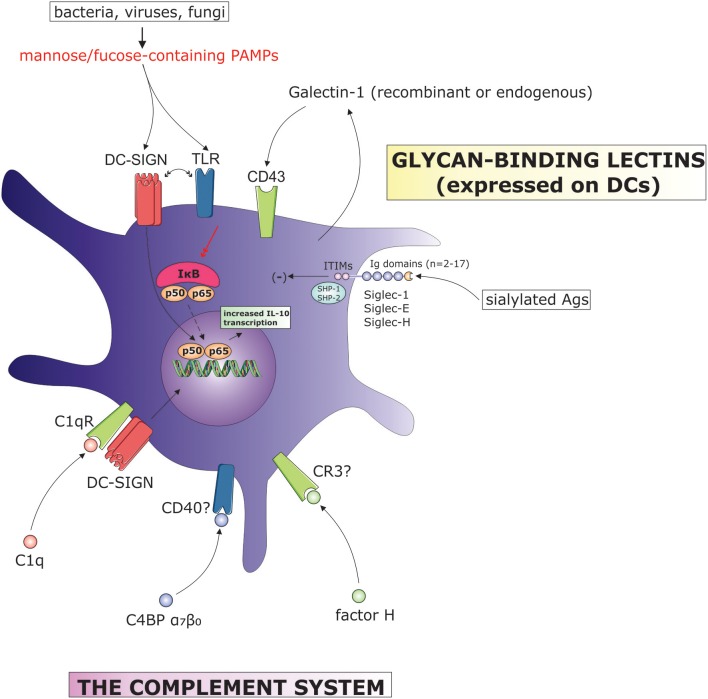
The influence of complement system components and surface-bound lectins on DC tolerogenic function. (C4BP, C4b binding protein; CR, complement receptor; DC-SIGN, dendritic cell-specific ICAM-grabbing non-integrin; IL, interleukin; ITIM, immunoreceptor tyrosine-based inhibitory motif; IκB, inhibitor of κB; PAMPs, pathogen-associated molecular patterns; SHP, Src homology region 2 domain-containing phosphatase; TLR, toll-like receptor).

The recognition of specific glycan structures by endogenous lectins expressed on DCs play a major role in the shaping of the DC activation state and subsequent tailoring of adaptive immune responses. Although a great number of glycans with DC immunomodulatory properties are present on invading pathogens including bacteria, fungi or viruses, and therefore do not represent endogenous biomolecules, we will discuss this subject due to the importance of DC-expressed lectins. Considering their involvement in the immunoregulation of DC function, we will focus on three major lectin families, namely the galectins, siglecs, and C-type lectins (Figure 2).

### C-type lectins

C-type lectin receptors (CLRs) were initially described as scavenger receptors, however it is now clear that many of them can function as signaling, adhesion, as well as Ag receptors. The most representative CLR found on DCs is most likely the dendritic cell-specific intercellular adhesion molecule-3-grabbing non-integrin (DC-SIGN) (63). DC-SIGN is predominantly expressed on myeloid DCs, but has also been detected on interstitial DCs, dermal DCs, as well as monocyte-derived DCs. Its recognition of Ags proceeds via mannose- and fucose-containing glycans, present on various bacteria (*H. pylori, M. tuberculosis*), viruses (HIV-1, Ebola), fungi (*C. albicans, A. fumigatus*), and others. Signaling via DC-SIGN upon ligand binding has been extensively studied and associated with modulation of concomitant signaling via other pattern-recognition receptors (PRRs), such as TLRs. Simultaneous signaling via DC-SIGN and TLRs (e.g., TLR-3, TLR-4, or TLR-5) can result in increased IL-10 production by DCs, contributing to the pathogen-mediated deviation of adaptive immune responses from Th1 to Th2 (64). For example, *Mycobacterium scrofulaceum* can induce a semi-mature DC phenotype by simultaneously interacting with DC-SIGN and TLR-4. In this way, the pathogen exerts an immunoregulatory effect characterized by the DC's low co-stimulatory molecule expression level and simultaneous high expression level of PDL-2 and IL-10 (65). In a similar manner, *Lactobacillus rhamnosus* can induce a tolerogenic DC phenotype by simultaneously triggering DC-SIGN and TLR-2 signaling (66). Such DCs can subsequently induce autologous T cell differentiation toward FoxP3-expressing Tregs. However, the complex nature of ligand binding by DC-SIGN coupled with intricate PRR-signaling mediated interactions, does not always issue a tolerogenic outcome, but can also result in positive DC activation as well (67).

The common feature of pathogens that bind DC-SIGN is that by using above described mechanisms, they cause chronic infections by deviating the immune response away from Th1 development, which in turn allows their persistence (68). This is of course due to intracellular nature of these pathogens. Probably the most notorious example of pathogen evasion is that exerted by HIV-1, where the native function of an endogenous biomolecule is exploited for viral infection and immune suppression. Binding of HIV-1 to DC-SIGN forms a stable complex whereby after internalization, a small amount of virus can survive and stays protected from the immune system. Adding to this the suppression of DC function by DC-SIGN signaling after interaction with e.g., viral envelope protein gp120 (69), the chance of viral persistence and subsequent infection of T cells is strongly increased (63, 70).

### Galectins

The tolerogenic DC function mediated by IL-27 is also regulated by Galectin-1 (Gal-1), a glycan-binding protein encoded by the *lgals1* gene (71). Recently, Gal-1 has been recognized as having a central role in the resolution of various acute and chronic inflammatory conditions involving autoimmune diseases, allergic inflammation, cancer and infection (72). On the cellular level, Gal-1 mediates regulatory programs in both innate and adaptive immunity. It is secreted by a number of cells including macrophages (73), dendritic cells (74), activated lymphocytes (75, 76), Fox P3^+^ Tregs (77), and others. In an effort to demonstrate its association with TolDC function, Ilarregui et al. treated immature DCs with recombinant Gal-1 and found they exhibit important TolDC characteristics (74). The binding of Gal-1 to immature DCs resulted in membrane segregation of its proposed glycoreceptor, a leukosialin protein also known as sialophorin or CD43. Furthermore, TolDCs generated with Gal-1 or other tolerogenic stimuli such as IL-10, vit D_3_ or VIP displayed an increased expression of Gal-1. Using an *lgals1* knockout model, they highlighted the importance of Gal-1 expression in DC tolerogenic function, including the capacity to produce low levels of IL-12p70 and increased IL-27. The production of IL-27 by Gal-1-treated DCs was associated with a regulatory circuit involving increased differentiation of IL-10-secreting T cells (74).

Like DC-SIGN, Gal-1 is commonly distributed at sites of inflammation and pathogen entry. Additional similarities regarding its role in pathogen evasion have been recently demonstrated in a model of *Trypanosoma cruzi* infection (78). Using Gal-1-deficient mice, Poncini et al. have demonstrated a reduced mortality and lower parasite load compared to wild type (WT) mice. The absence of Gal-1 resulted in increased cytotoxic CD8^+^ T cell response compared to a more pronounced Treg response in WT mice. The observed tolerogenic circuit in the presence of Gal-1 was associated with increased tolerogenicity of Gal-1-expressing DCs (78). The broad immunoregulatory role of Gal-1 extends to other immunopathological scenarios, such as cancer and involves modulation of other immune cell types, namely macrophage polarization, eosinophil and neutrophil migration, Treg expansion and others (72), which falls beyond the scope of this manuscript. However, its important immunoregulatory role including that in context of DC tolerogenicity makes Gal-1 an increasingly important therapeutic target, which could be addressed with the aim to regulate immunity in various immune-mediated diseases.

### Siglecs

Siglecs are sialic acid-binding immunoglobulin-like lectins expressed on various immune cell types. Like other members of the Ig superfamily, they act as cell-surface transmembrane receptors and consist of 2–17 extracellular Ig domains (79). Most intercellular domains of Siglecs contain immunoreceptor tyrosine-based inhibitory motifs (ITIMs) which, contrary to activation motifs (ITAMs), deliver negative signals by recruiting SH2 domain-containing tyrosine phosphatases such as SHP1 and SHP2. Like C-type lectin receptors, Siglecs expressed on DCs can modulate TLR-mediated immune responses when bound e.g., by endogenous glycans. This was directly demonstrated with murine DCs, where stimulation of TLRs induces the expression of Siglec-E via the Myd88-dependent pathway (80). Increased expression of Siglec-E inhibited the activation of Nf-κB after TLR activation, which coincided, furthermore, with the inhibition of IFN-β and RANTES production, usually up-regulated during viral infection. In this manner, Siglec-E serves as part of a negative feedback loop maintaining homeostasis after infection. Signaling via intracellular ITIM domains of Siglec-E was associated with the inhibition of Nf-κB. In macrophages, signaling via ITIM domains of Siglec-9 resulted in increased IL-10 production after TLR-engagement by either LPS, peptidoglycans, unmethylated CpG, or double-stranded RNA (81). The tolerogenic role of DC-expressed Siglecs could also serve a therapeutic role. The initiation of immune responses by targeting Ags to Siglec-H expressed on plasmacytoid DCs was shown to result in the inhibition of T cell responses during disease progression in an EAE model (82). Delivery of myelin-derived auto-Ag to plasmacytoid DCs subsequently induced T cell hyporesponsiveness and reduced polarization toward Th1/Th17 effector T cells. Also in humans, a subset of plasmacytoid DCs expressing Siglec-1 and additionally characterized by high HLA-DR and CD11c expression levels, displayed a predominantly semi-mature phenotype. In contrast to Siglec-1-negative pDCs, Siglec-1-positive pDCs do not produce type I IFNs upon engagement of TLR-7 or TLR-9 (83). The induction of tolerance initiated by Siglec-mediated Ag up-take can expand beyond the inhibition of T cell activation to *de novo* induction of regulatory T cells. In a recent study, Perdicchio et al. pulsed splenic CD11c^+^ DCs with sialic acid-modified Ags. When these Ag-loaded DCs were used in co-cultures with naïve T cells there was a 2- to 5-fold increase in FoxP3^+^ T cell populations. The induction of TolDCs by up-take of sialylated Ags was shown to be mediated mainly via Siglec-E, as demonstrated using *siglec-e*^−/−^ models (84).

## The complement system

The complement system is part of the innate immune system which consists of more than sixty protein components that involve activation fragments, proteases and enzymes, receptors for complement effector molecules, as well as regulators and inhibitors (85). Complement activation releases cleavage products that bind a number of receptors found on myeloid, lymphoid and stromal cells (86). These complex interactions provide a basis for a link between certain complement proteins and the control of adaptive immune responses, as first described by Fearon and Carter (87). As discussed below, DCs play a central role in this type of regulation (Figure 2). One of first demonstrations regarding the role of the complement system in DC regulation was described by Castellano and colleagues, who showed that differentiation of DCs from monocytes in the presence of C1q generates CD1a^+^, DC-SIGN^+^ DCs resistant to LPS-induced maturation with low co-stimulatory and allo-stimulatory potential (88). This mechanism was also observed in recognition of apoptotic cells. The tolerogenicity triggered by apoptotic cells is a well described mechanism of peripheral tolerance, which allows continuous clearance of dead cells without collateral inflammatory damage. Interestingly, DCs that ingest apoptotic cells which are bound to C1q, attain greater tolerogenic function compared to DCs exposed to apoptotic cells alone(89). This was evident by increased PDL-2/CD86 ratio and the capacity to induce Th1 responses. Such evidence could have important implications in resolving the underlying mechanisms of existing cell therapies, namely the extracorporeal photopheresis, where T cell apoptosis is speculated to be of major importance for its tolerogenic effect in e.g., GvHD treatment (90). The receptor for C1q, namely the C1qR, was shown to mediate its cell signaling events through DC-SIGN by forming a membrane complex consisting of C1q, C1qR, and DC-SIGN (91). In this manner, C1qR utilizes the intracellular signaling domain of DC-SIGN.

More recently, complement regulators and inhibitory proteins were described as having an important immunosuppressive role in terms of DC modulation. One isoform of C4b-binding protein (C4BP), an important soluble inhibitor of the classical and lectin pathways of complement activation, namely C4BP α_7_β_0_ isoform, can induce a tolerogenic state in monocyte-derived DCs (92). There are several ligands that C4BP can bind, for example, other complement proteins, CD91, heparin, etc. However, it has been proposed that C4BP also represents an alternative ligand for CD40, which is abundantly expressed on DCs to serve as a co-stimulatory receptor (93). Whether CD40 is the primary receptor for C4BP on DCs in this context, or if there are other ligands involved, remains to be determined. After the activation of DCs pre-treated with C4BP α_7_β_0_ isoform, the cells obtained a semi-mature state with low expressions of CD80, CD83, and CD86 molecules. They were also unable to produce pro-inflammatory cytokines, such as TNF-α, IL-12, etc. but showed increased production of IL-10. In terms of function, they were incapable of inducing allogeneic T cell proliferation and promoted the polarization of CD4^+^CD25^+^CD127^low^FoxP3^+^ T cells. Although in the physiological state, the ratio of isoforms C4BP α_7_β_1_ vs. C4BP α_7_β_0_ is roughly 4:1, under acute phase responses C4BP α_7_β_0_ can drastically increase, which could have important implications in terms of DC function (94).

First characterized in 1965, factor H (FH) is a major regulator of the alternative pathway of complement activation (95). It is mainly synthesized in the liver but for the exception of certain tissue cells such as endothelial cells, glomerular mesangial cells, mesenchymal stem cells, and others (96–98). Besides the direct negative regulation of complement activation, FH also interacts with other endogenous ligands or receptors which extends its activity to protection from oxidative stress (99), modulation of platelet function (100), and regulation of immunity via interaction with several cells of the immune system (101, 102). Recently, treatment of DCs with FH during the early stages of differentiation from monocytes was shown to have an important influence on their tolerogenic capacity (103). When used in 10 μg/ml concentration, factor H caused an extensive down-regulation of all major co-stimulatory molecules, namely CD40, CD80, and CD86, as well as MHC class II molecules. The expression of inhibitory surface molecules was not confirmed, however, FH-treated DCs produced low quantities of pro-inflammatory cytokines and allowed for increased IL-10 production. Assessment of the DCs' functional capacity revealed a low allo-stimulatory capacity. Activation of responsive T cells in co-cultures resulted in down-regulation of IFN-γ-producing T cells and an increase in the induction of FoxP3 expression, where FH-treated DCs were used as stimulators. Interestingly, the group found that the same surface binding-region of the FH protein which binds to C3b and glycosaminoglycans on host cells to mediate cell-surface protection, is also responsible for inducing the tolerogenic effect on DCs (103). Although the corresponding receptor on DCs responsible for mediating the described tolerogenic effects was not determined, the authors excluded the involvement of interaction between FH and CR3, which has been reported on many occasions in the past (104, 105).

Interestingly, the production of FH can also be induced by DCs themselves via the influence of local microenvironment. This effect was shown to be characteristic of TolDCs, which are induced by specific factors, particularly IFN-γ. A similar effect by DCs was also observed for IL-12 family member IL-27. In addition to the above described tolerogenic properties of both IFN-γ and IL-27, the induction of FH by DCs complements the tolerogenic activity of these cytokines (106). In this manner, increased production of FH by either IFN-γ or IL-27-stimulated DCs resulted in the decreased capacity to stimulate T cell proliferation and deviation from Th1 polarization. The possibility of autocrine tolerogenic effects of DC-secreted FH is not excluded.

The above mentioned contributions of complement cleavage products to induction of DC tolerogenicity expand the classical view of the role of complement cascade within the innate immune system. By additionally serving as soluble mediators, capable of inducing immune cell signaling via receptor-ligand binding, the role of complement activation extends to regulation of T cell immunity either via suppression of effector T cells or *de novo* induction of Tregs. It is of importance that the extent of this contribution *in vivo* is even further elaborated in the future.

## Growth factors

Both haematopoietic and growth factors from other tissues can importantly govern the development and biological function of DCs (Figure 1). In the mid-1990s, Gabrilovich et al. identified a soluble factor released from tumor cells that can dramatically affect DC maturation. This was recognized as vascular endothelial growth factor (VEGF) (107). Today VEGF is well recognized as a major pro-tumorigenic growth factor, which also represents an important therapeutic target (108). VEGF binds its corresponding Flt-1 (VEGFR1) and VEGFR2 receptors expressed on DCs and can negatively affect maturation via inhibition of the Nf-κB pathway (109). While VEGFR1 can be found both on immature and mature DCs, the expression of VEGFR2 was reported only on mature DCs (110). Although the effect of VEGF on DC co-stimulatory molecules is less pronounced, it extensively suppresses the T cell-stimulatory capacity of DCs (111). In patients with chronic obstructive pulmonary disease, increased concentrations of VEGF have been positively correlated with reduced CD83 expression on DCs and the number of immature DCs was significantly related to the severity of disease (112). Recently, a study by Nougboli et al. examined the role of neuropilin-1 (Nrp-1), another known receptor of VEGF. Neuropilin-1, also known as BDCA-4 or CD304, is found primarily on plasmacytoid DCs in humans, while in mice it can be detected on myeloid, bone marrow-derived DCs (BMDCs). It has been demonstrated that in BMDCs, Nrp-1 interacts directly with TLR-4, and suppresses down-stream signaling upon LPS-induced maturation (113). In this way, Nrp-1 causes extensive inhibition of ERK and Nf-κB pathways and consequent down-regulation of MHC class II and co-stimulatory molecules, along with pro-inflammatory cytokine production.

Another member of the VEGF family, namely the placental growth factor (PIGF), has also been designated as having a DC-modulatory capacity. The differentiation of DCs from monocytes in the presence of PIGF resulted in alternative responses to LPS-induced stimulation. Such DCs were characterized by low expression levels of CD40, CD80, CD83, and CD86 (114). Production of IL-8, IL-12p70, and TNF-α was also down-regulated, as was the expression of MHC class II molecules. Interestingly, PIGF did not seem to inhibit monocyte-to-DC differentiation as CD1a expression was increased in treated cultures. Similarly to VEGF, PIGF inhibits Nf-κB activation and its effects could be reversed by anti-VEGFR1 mAb, demonstrating its similarity to VEGF's mode of action. However, PIGF seems to affect co-stimulatory molecule expression more extensively. In terms of its tolerogenic role, PIGF seems to bear certain similarities with IFN-γ. While its immunosuppressive effects on DCs have been demonstrated when acting alone, PIGF has also been shown to enhance TLR-mediated signaling. Bagby and colleagues demonstrated PIGF can amplify TLR-dependent gene expression via MAPK-activated protein kinase-2 pathway, when DCs were stimulated with R848, a TLR-8 ligand (115). Therefore, PIGF can also cause exaggerated inflammatory response, depending on pathogen presence.

Hepatocyte growth factor (HGF) was first described as an important mitogen for a number of epithelial and endothelial cells involved in organ regeneration, such as liver regeneration by stimulating the proliferation of hepatocytes (116, 117). It can also act on different types of immune cells such as hematopoietic stem cells, monocytes, macrophages, and dendritic cells via its corresponding proto-oncogenic c-Met receptor, also known as HGF receptor (HGFR) (118). A little more than 10 years ago, two studies published within the span of a year, first described that differentiation of monocytes to dendritic cells in the presence of HGF or its administration to isolated native DCs leads to development of high IL-10-, low IL-12-producing dendritic-like cells (119, 120). Dendritic cells treated with HGF also showed an increased expression of ILT-3, commonly associated with DC tolerogenicity. Interestingly, blockade of ILT-3 or IL-10 using neutralization mAbs partially restored the proliferation of FoxP3^+^ T cells induced by HGF-treated DCs. Systemic treatment with HGF was shown to ameliorate autoimmune diseases in models such as EAE (121). In this context, the main cellular mechanism was the increased generation of TolDCs via induction of the glucocorticoid-induced leucine zipper (GILZ), which is also the underlying mechanism for TolDC-induction by IL-10 or glucocorticoids (122). In the inflamed c(CNS) environment during EAE, HGF-induced TolDCs can contribute to the increased presence of IL-10-secreting, FoxP3^+^ Tregs (123). The last to be mentioned in terms of having a capacity to induce DCs with tolerogenic properties is adrenomedullin (AM), a hormone first described with hypotensive properties and found in adrenal medulla, which gave rise to its current nomenclature (124). Now known to be ubiquitously present in all biological fluids and several tissues, the levels of AM are up-regulated during inflammation, infection and are inducible by immunological stimuli such as IL-1 and TNF-α (125). Besides being a potent vasodilator, AM was shown to have an important influence on the differentiation (126), proliferation (127), migration (128), and apoptosis (129) of various cells. Adrenomedullin was also shown to possess immunomodulatory properties *in vivo* by attenuating leukocyte recruitment and inhibiting cytokine production (130). Dendritic cells have been shown to express AM receptors, which consist of calcitonin receptor-like receptor (CRLR), as well as AM1 and AM2 receptors (131). The treatment of BMDCs with 10^−7^–10^−6^ M concentrations resulted in a semi-mature DC phenotype with tolerogenic properties. Stimulation with AM induced IDO-competence in treated DCs and promoted their ability to induce FoxP3 expression in responding T cells. When studied in a model of EAE, systemic treatment with AM attenuated disease severity and incidence of EAE, characterized by reduced demyelination and axonal damage (132). Importantly, AM increased the number of IL-10-producing T cells with suppressive effect on disease development. Within the same study, TolDCs obtained *ex vivo* using AM, reduced the severity of EAE following their administration *in vivo*. Such DCs were characterized by low expression levels of CD40 and CD80, low production of TNF-α and IL-12, and with an induced production of IL-10 (132).

## Hormones

**Figure 3:**
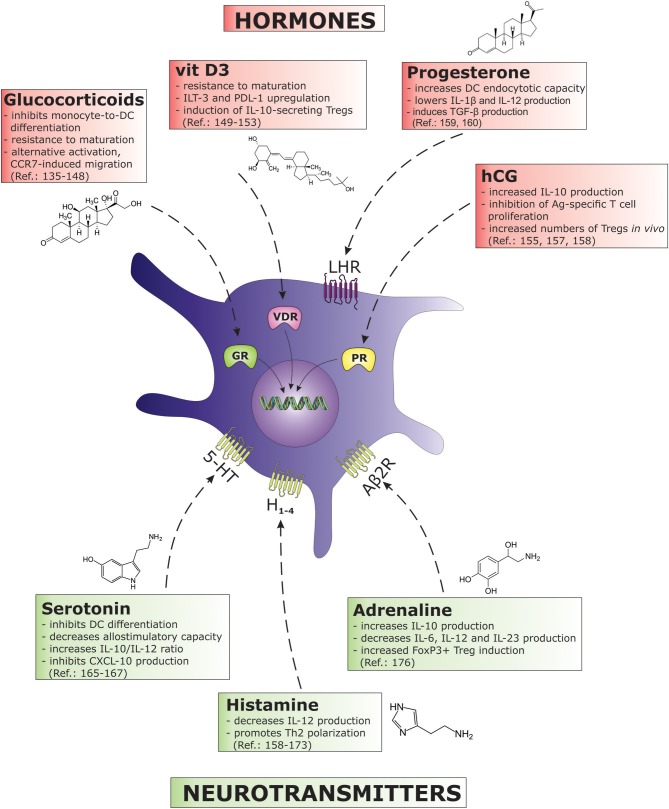
The figure describes various hormones and neurotransmitters and their immunosuppressive effects on DC biology. Each biomolecule as associated with its correspondent receptor on DCs. (5-HT, 5-hydroxytryptamine (serotonine) receptor; Aβ2R, adrenergic β2 receptor; GR, glucocorticoid receptor; H, histamine receptor; hCG, human chorionic gonadotropin; IL, interleukin; ILT, immunoglobulin-like transcript; LHR, luteinizing hormone/choriogonadotropin receptor; PDL, programmed death ligand; PR, progesterone receptor; TGF, transforming growth factor; VDR, vitamin D receptor).

The bidirectional interactions between the endocrine and the immune system have been demonstrated over many decades and are well accepted by the scientific community (133). Immune cells, namely DCs, carry receptors for numerous hormones that importantly influence the cell cycle and differentiation (Figure 3). The most well established TolDC-inducing hormones of all are perhaps steroid hormones, particularly glucocorticoids (GCs), which bind to the glucocorticoid receptor in the nucleus. In addition to naturally occurring GCs (134), the effects of various synthetic glucocorticoids have been studied in the context of TolDCs in the past, including methylprednisolone (135), prednisolone (136, 137), prednisone (138), and mostly dexamethasone, presumably because of its potency (139–141). Glucocorticoids strongly inhibit monocyte-to-DC differentiation leading to a CD1a^low^CD14^+^CD16^+^ phenotype. This effect is associated with the suppressed expression of co-stimulatory and Ag-presenting molecules (CD40, CD54, CD86) and the inhibition of maturation marker CD83 after DC activation (142). Dexamethasone induces tolerogenic DC characteristics administered either during the differentiation phase, or to cultures of already established DCs. Immature DCs treated with dexamethasone are resistant to maturation. When such DCs are simultaneously exposed to maturation stimuli (e.g., LPS) they can become “alternatively activated,” possessing certain features of mature DCs like migration via the CCR7 (143). Migration toward secondary lymph nodes in response to CCL19 or CCL21 would represent a greater probability of interacting with responsive T cells, a welcome characteristic in the design of negative DC vaccines for the treatment of autoimmune or chronic inflammatory diseases. Indeed, dexamethasone is frequently used in protocols for the generation of clinically-applicable TolDCs with or without GMP-grade TLR agonists such as monophosphoryl lipid A (MPLA) (144). Dendritic cells treated with dexamethasone and MPLA possess strong regulatory functions, and have been shown to modulate both naïve and memory T cell responses (145). As a further boost to its tolerogenic potential, dexamethasone is also used in combination with other immunosuppressive drugs like minocycline (146) or more frequently with another naturally occurring secosteroid hormone, vit D_3_ (147).

The hormonally active metabolite of vitamin D, calcitriol, or vit D_3_ is, like dexamethasone, one of the most thoroughly studied TolDC-inducing agents. Its efficiency in inducing DC tolerogenicity was recognized around the same time as that of dexamethasone, however it induces certain unique TolDC characteristics. For instance, similarly to IL-10, vit D_3_ upregulates ILT-3, an inhibitory molecule strongly associated with TolDCs (148–150). Besides ILT-3 being the denominator of tolerogenic DC state, the functional importance of its expression on vit D_3_ -treated DCs has not been directly confirmed so far. On the other hand, the blockade of PDL-1, which is also extensively induced upon vit D_3_ treatment, has been shown to be important (151). Neutralization of PDL-1 in DC-T cell co-cultures resulted in an increase of IFN-γ-producing T cells and decreased the percentage of IL-10-secreting Tregs. Vitamin D_3_ exerts its actions after binding to its corresponding nuclear receptor, the vitamin D receptor (VDR). Recently, the mechanisms of vit D_3_ -mediated tolerance induction in DCs have been associated with the modulation of cellular metabolic pathways. Using microarray analysis it has been shown that vit D_3_ upregulates several genes directly associated with glucose metabolism, that is, Krebs cycle and oxidative phosphorylation (152). More specifically, the availability of glucose and glycolysis, regulated by the PI3K/Akt/mTOR pathway, was demonstrated to have direct control over the tolerogenic DC phenotype and function induced by vit D_3_.

During human pregnancy, an increased number of leukocytes, including DCs, can be found in the pregnancy decidua and the weight of evidence supports the role of sex hormones in DC regulation (153–155). Human chorionic gonadotropin (hCG) is an early pregnancy-associated hormone and plays a vital part in pregnancy establishment and maintenance. Human CG has been shown to bind respective receptors on DCs, namely the luteinizing hormone receptor (LHR), and can stimulate the production of immunosuppressive IL-10, thereby inhibiting Ag-specific T cell proliferation (154, 156). In a mouse model, the adoptive transfer of hCG-treated DCs prior to mating had a protective effect on pregnancy stability, and was accompanied by increased Treg numbers and decidual expression of TGF-β and IL-10 (157). A number of studies also support the immunosuppressive function of progesterone, which was shown to regulate the DC stimulatory capacity in rats (158). While estrogen has been shown to promote DC differentiation (159), high physiological concentrations of progesterone that can be seen during pregnancy (cca 10^−6^ M) have recently been demonstrated to counteract this effect (160). In contrast to estrogen, progesterone increased the endocytotic capacity of DCs and significantly lowered IL-12 and IL-1β, while increased TGF-β production after LPS-induced maturation.

## Neurotransmitters

The nervous system plays an important role in communicating and interacting with the immune system. The ability of immune cells to respond to released neurotransmitters is reflected in immunological changes that accompany psychological disorders. For example, immune suppression followed by increased inflammatory activity has been found in depressed patients (161, 162). Several neurotransmitters exert their immunomodulatory effects by influencing DC biology (Figure 3). The immunomodulatory characteristic of serotonin (5-HT), a neurotransmitter closely related to depression, has been demonstrated by its direct effects on PBMCs, differentially modulating cytokine production via the 5-HT_2A_ receptor (163). In terms of DCs, 5-HT has been shown to have a potent effect on various aspects of their biology. In the presence of 5-HT, monocytes differentiate into DCs with reduced expression levels of CD1a and co-stimulatory molecules, while retaining their expression levels of CD14 (164). Functionally, such DCs displayed a decreased allo-stimulatory capacity and increased IL-10/IL-12 ratio. The use of specific 5-HT receptor antagonists suggested the involvement of receptors 5-HTR_1_ and 5-HTR_7_ in these immunoregulatory actions. Serotonin was also associated with the modulation of DC migratory capacity. Modulation of chemokine production was characterized by the inhibition of the Th1 chemoattractant CXCL-10, and the up-regulation of CCL-22, which primarily attracts Th2 effector cells (165). In this same study, the addition of 5-HT during DC maturation increased the IL-10/IL-12 ratio and up-regulated the production of IL-6. In terms of function, 5-HT-treated DCs favored the induction of Th2 over Th1 responses. Very recently, a detailed analysis on 5-HT1-7 expression and function on CD1a^+^ and CD1a^−^ monocyte-derived DCs (MoDCs) was performed (166). The authors found an important role for HT_2B_ expression on CD1a^+^ MoDCs, wherein the activation of this receptor strongly inhibited DC stimulation via TLR-2, TLR-3, and TLR-7/8. They found a strong down-regulation of cytokine and chemokine expression such as TNF-α, IL-6, IL-8, IL-12, and CXCL-10. The resulting DC phenotype deviated from the capacity to induce Th1 and Th17 polarization of T cell responses. The expression of 5-HT_2B_ was not detected on CD1a^−^ populations and was highlighted as an important negative regulator of inflammatory responses mediated by CD1a^+^ MoDCs.

Another monoamine neurotransmitter, histamine, has been characterized as having numerous immunoregulatory functions including some exerted on DCs. In this context, the effect of histamine can be interpreted as anti-inflammatory in the sense that it was initially described as having the capacity to skew DCs toward Th2 polarization. Dendritic cells treated with histamine showed a dose-dependent down-regulation of LPS-induced IL-12 production (167). When co-cultured with naïve CD4^+^ T cells, such DCs promoted an induction of IL-4-secreting T cells. Using synthetic antagonists, the negative effect of histamine on IL-12 production was found to be mediated via H1 and H2, and also by H3 and H4 receptors (168, 169). In a recent study, histamine-dependent cytoskeleton re-organization was found crucial for its known Th2 polarizing effect (170). Indeed, the connection between cytoskeletal arrangement and DC polarizing capacity has been demonstrated before (171). Aldinucci et al. have shown that histamine affects the mature cytoskeleton of TLR-4-stimulated DCs, but not when the cells are matured with TLR-2 agonists, suggesting the association between various maturation states achieved via different TLR pathways and differences in corresponding cytoskeletal rearrangement. As shown by this group, as well as others, histamine also down-regulates CXCL-10 expression by DCs (172). On the other hand, the up-regulation of IL-10 production by histamine-treated DCs has not been confirmed in all studies. Interestingly, in a recent study using the EAE model, disease symptoms were alleviated by immethridine, a selective H3 agonist. The mechanism mainly proposed was the inhibition of DC function, low co-stimulatory molecule expression, as well as the inhibition of Nf-κB p65 (173).

Both the sympathetic and parasympathetic nervous systems are known to affect inflammatory responses via released adrenergic and cholinergic mediators (174). Nijhuis et al. have compared the effect of acetylcholine (ACh), nicotine (both parasympathetic), and epinephrine (sympathetic) on various DC functions such as maturation, cytokine production, endocytosis and function (175). They demonstrated that adrenergic signals, rather than cholinergic, enhance immunosuppressive DC characteristics. Activation of the adrenergic β2 receptor (Aβ2R) by epinephrine enhanced IL-10 production by DCs and at the same time decreased the production of IL-6, IL-12, and IL-23. Such DCs were potent at inducing Fox P3^+^ Tregs with a suppressive capacity. The tolerogenic potential of Aβ2R-stimulated DCs was not dependent on the autocrine actions of released IL-10, TGF-β, or retinoic acid secretion, but rather on direct signaling via Aβ2R itself. Dendritic cells are also known to express both muscarinic and nicotinic receptors. In another study by Gori et al., treatment of MoDCs and directly isolated CD1c^+^ blood DCs with ACh stimulated the surface expression of OX40L, a Th2-promoting co-stimulatory molecule and Th2-associated chemokine CCL22 (176).

## Conclusion

The data summarized in this review show a definite impact of various endogenous biomolecules on induction of DCs' tolerogenic state. The importance of tissue microenvironment, as well as immunomodulatory factors produced by immune cells themselves, has been proposed as a crucial player in regulating both innate and adaptive immune responses many years ago in so-called “danger model” proposed by Polly Matzinger. It is now becoming increasingly clear that both tissues and immune cells, via soluble or surface-bound factors, can regulate the immune system in both ways, leading to increased activation or on the other hand, predominantly tolerogenic immunological outcomes. Since DCs serve a central role in bridging innate and adaptive immunity, understanding the manner in which they are influenced by the above mentioned repertoire of diverse factors can be regarded as fundamental to interpretation of their functionality toward cells of the adaptive immune system.

## Author contributions

UŠ designed and organized the manuscript as well as wrote several topics within the article. PR wrote selected topics within the article and co-organized the manuscript.

### Conflict of interest statement

The authors declare that the research was conducted in the absence of any commercial or financial relationships that could be construed as a potential conflict of interest.
